# Routes for Potassium Ions across Mitochondrial Membranes: A Biophysical Point of View with Special Focus on the ATP-Sensitive K^+^ Channel

**DOI:** 10.3390/biom11081172

**Published:** 2021-08-08

**Authors:** Yevheniia Kravenska, Vanessa Checchetto, Ildiko Szabo

**Affiliations:** Department of Biology, University of Padova, 35131 Padova, Italy; k.evgeniya.v@gmail.com (Y.K.); vanessa.checchetto@unipd.it (V.C.)

**Keywords:** mitochondria, ion channels, electrophysiology, ATP-dependent potassium channel

## Abstract

Potassium ions can cross both the outer and inner mitochondrial membranes by means of multiple routes. A few potassium-permeable ion channels exist in the outer membrane, while in the inner membrane, a multitude of different potassium-selective and potassium-permeable channels mediate K^+^ uptake into energized mitochondria. In contrast, potassium is exported from the matrix thanks to an H^+^/K^+^ exchanger whose molecular identity is still debated. Among the K^+^ channels of the inner mitochondrial membrane, the most widely studied is the ATP-dependent potassium channel, whose pharmacological activation protects cells against ischemic damage and neuronal injury. In this review, we briefly summarize and compare the different hypotheses regarding the molecular identity of this patho-physiologically relevant channel, taking into account the electrophysiological characteristics of the proposed components. In addition, we discuss the characteristics of the other channels sharing localization to both the plasma membrane and mitochondria.

## 1. Introduction

In 1961, Peter Mitchell formulated the chemiosmotic theory [1], according to which (i) electron transport through the respiratory chain of mitochondria promotes the flow of protons from the matrix into the intermembrane space, allowing the formation of a H+ gradient (protonmotive force), (ii) this process is directly related to the activity of F_1_F_o_-ATPsynthase and to the production/hydrolysis of ATP, (iii) the inner mitochondrial membrane (IMM) is impermeable to ions, including H^+^. Despite this assumption, the author predicted the necessity for ions to be transported through the IMM. Indeed, extensive research over the last few decades has highlighted an important role for potassium transporters/channels in the regulation of the IMM potential (Δψ), redox state and mitochondrial volume, as summarized by recent, detailed reviews, e.g., [2,3,4,5,6,7]. By modulating these important factors, K^+^ channels impact not only on bioenergetic efficiency and ATP production by mitochondria, but also on cellular signaling events such as, for example, apoptotic signaling [8,9], Wnt signaling [10], and cGMP-related pathways [11]. As a consequence, IMM K^+^ channels emerged as important players in the context of cancer [12], cardioprotection [13], and neuronal protection [9], and may even play a role in the inflammatory response [14]. Thanks to intensive research carried out by several excellent groups in the field, mitochondrial potassium channels are currently in the spotlight of the scientific community working on different pathologies.

## 2. Multiple Routes for Potassium across the Outer and Inner Mitochondrial Membranes

The passage of this ion, which is normally present at a concentration greater than 100 mM in the cytosol, follows the electrochemical gradient largely dictated by the very negative resting membrane potential across the IMM (around −180 mV). Mitochondria maintain the matrix K^+^ concentration at 150–180 mM [15]. The influx of K^+^ can occur through the mitochondrial channels, while the excess K^+^ matrix is expelled by the antiporter K^+^/H^+^. The consensus view is that the voltage-dependent anion channel isoforms (VDACs) of the outer mitochondrial membrane (OMM) [16,17] are sufficiently large to allow the flux of different metabolites and ions, including K^+^. Indeed, VDACs (VDAC1-3) show a slight cation selectivity in the partially closed state [18] and, although tightly regulated [19], they may ensure a continuous flux of potassium into and out of the mitochondrial intermembrane space, allowing an equilibrium between this compartment and the cytosol. It was surprising, therefore, that an inwardly rectifying K^+^ channel that was responsive to voltage, osmotic pressure and cAMP has been identified directly by patch clamping mitochondria isolated from the spinal cord [20]. The physiological importance of this channel is not clear, but importantly, this work confirmed the idea that VDAC may adopt a completely closed state across the OMM [21], since no VDAC-like current was observed by electrophysiology. It is of note that even though VDACs were shown to mediate calcium flux across the OMM [22,23], a small channel, namely a functional α7 nicotinic acetylcholine receptor, was able to regulate calcium flux across the OMM [24], suggesting that the OMM might indeed be not freely permeable to ions such as calcium and potassium.

In contrast to the OMM, the IMM harbors a number of potassium channels, and a K^+^/H^+^ exchanger. Given that several recent reviews dealt with the former topic (see also [5] in this Special Issue), here we only mention the main classes of K^+^ channels, that can allow the passage of only K^+^ (K^+^-selective channels) or of other cations as well (K^+^-permeable channels). In the former category, the following channels have been identified directly by patch clamping of mitochondria: (1) ATP-dependent potassium channel [25]; (2) voltage-gated shaker type K^+^ channel Kv1.3 [26,27]; (3) calcium-activated intermediate- conductance K^+^ channel (IK(Ca)) [28]; (4) calcium-activated big conductance potassium channel (BK(Ca)) [29,30]; (5) small-conductance calcium-activated K^+^ channel (SK(Ca)) [31]; (6) renal medullary channel ROMK [32]; and (7) two-pore potassium channel TASK-3 [33]. The hyperpolarization-activated, cyclic- nucleotide-gated channel isoforms HCNs belong to the latter category of potassium-permeable channels [34,35].

In most of the above studies, the mitochondrial channels recorded in the IMM highly resembled those of the plasma membrane (PM), at least regarding their conductance, which is a basic biophysical feature of all ion channels. In fact, our current understanding is that, for example in the cases of TASK-3, IK(Ca), SK(Ca), Kv1.3, and HCN, the same proteins residing in the PM locate to the IMM as well, giving rise to comparable channel activities. Although mitoplasts are well distinguishable from other organelles when performing patch-clamp experiments, more sophisticated methodologies that exclude the possibility of contamination are also available [36,37]. In most studies, multiple techniques were exploited to confirm dual localization of a given protein, such as Western blot to assess contaminations by other membranes, immunogold electron microscopy and modulation of mitochondrial activity and/or K^+^ uptake into mitochondria by pharmacological or genetic tools. Kv7.4 [38] and Kv1.5 [39] have been identified in the IMM of cardiomyocytes and macrophages, respectively, using biochemical/pharmacological tools only.

The mechanism of dual targeting is not known for most IMM K^+^ channels. With the exception of HCNs and ROMK2 (see below), bioinformatic tools do not predict mitochondrial localization due to the lack of a typical N-terminal mitochondria-targeting sequence. Our recent data indicate that, for example, in the case of Kv1.3, association of the channel with caveolin-1 promotes PM targeting, while the lack of such functional coupling causes accumulation of the channel in mitochondria, with severe consequences on mitochondrial function and cell survival [40]. Whether association with caveolin may play a role in channel trafficking and subcellular targeting also in the case of other K^+^ channels is an interesting point for future investigation. Another interesting candidate worth consideration is SKD3 (suppressor of potassium transport defect 3), also known as caseinolytic peptidase B protein homolog (CLPB), a broadly expressed member of the family of ATPases associated with diverse cellular activities (AAA+). A recent study highlighted that ATP controls the ability of SKD3 to self- associate or form complexes with other proteins in the intermembrane space of mitochondria [41]. Human CLPB contains an ankyrin-repeat domain, and, for example, channels of the Kv7 family harbor an ankyrin-binding domain [42]. Whether and how this IMS-located protein might regulate IMM K^+^ channel import/assembly/function or exert a quality control remains an open question.

Since patch clamping of mitoplasts (i.e., of mitochondria devoid of the OMM) is technically demanding, unfortunately a complete biophysical and pharmacological characterization has not been carried out in all cases, thus hampering a strict comparison of the PM-located channels with those of their IMM counterparts. Nevertheless, as illustrated in Table 1, a relatively good match has been found in many cases, at least regarding the conductance values. It is of note that for some of the mentioned channels, the range of described conductance values is quite wide, which can be due to several factors. First of all, the composition of the working solutions (in particular, by the concentration of Ca^2+^ and K^+^) and the type of cells/tissues/organisms are not the same in each work. Depending on the cell type, the general composition and characteristics of the channels may differ, for example, due to lack of interaction with specific receptors/regulatory subunits. Within the same family of channels, differences in conductance can be explained by alternative splicing, post-translational modifications, as well as by the homo- and heteromerization of the channel [6]. The protocol applied to elicit channel activity is also important, since a wider range of voltages makes it possible to determine the conductance with greater accuracy.

Important questions are still unanswered, in particular regarding Kv channels that are normally active in the PM at depolarizing voltages. Thus, the factors allowing these channels to operate at the very negative IMM potential (Δψ, around −160 to −180 mV) remain to be determined. Although the fact that, e.g., Kv1.3 is active in the IMM is indicated by changes in Δψ upon its inhibition using specific blockers [26], recent studies pointed to its involvement in the apoptotic cascade via its interaction with complex I [43] and to its role in linking respiration to proliferation, not necessarily relying on Kv1.3 ability to mediate K^+^ flux [44]. It is interesting to note that other mitochondrial potassium channels are also physically and/or functionally coupled to respiratory chain complexes: mitoBK (Ca) functionally and physically interacts with complex IV [45], mitoKATP function has been linked to complex II [46,47], ROMK2 is associated with complex V [48], while TASK-3 [49] and HCN [34] also seem to interact with complex V. It is tempting to speculate that the specific association of different K^+^ channels with distinct OXPHOS complexes may allow a reciprocal fine-tuning of their activities.

**Table 1:** Comparison of single-channel conductance values of the plasma membrane- and the inner mitochondrial membrane-located K^+^ channels obtained by the patch-clamp technique (in excised inside-out or mitoplast-attached configurations). Open probabilities are also reported, where available.

Channel	Conductance of PM Channel	Conductance of Mitochondrial Channel
***ATP-dependent K^+^ channel***	13–68 pS in 140 mM KCl [50,51] 20–80 pS in 140 (bath)/5.4–100 (pipette) mM KCl [52] ~80 pS in 145 mM K^+^ [53,54] 135 pS in 120 (bath)/60 (pipette) mM K^+^ [55]	From 10 to 100 pS in 150 mM KCl (see Table 2).
	*Open probability:* *NPo ~0.32 at −100 mV* [51] ≈*0.9 between −100 and 60 mV [54]*	*Open probability:* *0.74 at 40 mV [56] 0.57 at −50 mV [32] 0.24 at −60 mV [57]*
***Kv1.3***	24 pS in 140 mM KCl [58]	~25 pS in 134 mM K^+^ [26] 109 pS in 150 mM KCl [27]
	*Open probability:* *~0.013 at 50 mV [58]*	*Open probability:* *0.5 at −60 mV to 0.75 at 60 mV [27]*
***BK(Ca)***	~180 pS in 143 KCl [59] 187 pS in 144 KCl [60] 260–293 pS in 150 KCl [61] 250 to 300 pS in 150 mM K^+^ (e.g., [62])	190 pS in 130 (bath)/10 (pipette) mM K^+^ [63] 145 to 307 pS in 150 mM K^+^ [11,29,64,65,66,67,68]
	*Open probability:* *~0.27 at −40 mV [61]*	*Open probability:* *0.79 at 80 mV [63] 0.5 at −33 mV [29]* ∼*0.16 at −60 mV to* ∼*0.94 at 60 mV [65]* ∼*0.54–0.9 at 60 mV [11,66]* ∼*0.25–0.76 at −40 mV [67,68]*
***IK(Ca)***	~25 pS in 150 (bath)/140 (pipette) mM KCl [69] 31 pS in 160 mM K^+^ [70] 33–34 pS in 130 (bath)/145 (pipette) mM KCl [71] 39 pS in 120 mM K^+^ [72]	10 to 90 pS in 150 mM KCl [28]
	*Open probability:* *0.6 and 0.4 at −50 and 50 mV, respectively [69]* *<0.5 between −120 and 60 mV [70]* *0.021 at −60 mV, 0.013 at −20 mV [71]*	
***SK(Ca)***	8 pS in 200 (bath)/4 (pipette) mM KCl [73] 15 pS in 140 mM KCl [74] 40–50 pS in 140 mM KCl (pipette) [75]	Not determined at single channel level (for whole-mitoplast recording, see [31])
***ROMK***	~30 pS in 5 (bath)/140 (pipette) mM KCl [76] 39 pS in 145 K^+^ mM [77]	94 pS in 150 mM KCl [32]
	*Open probability:* *0.88 between −40 and −80 mV [76]* *0.82 at −60 mV and 0.92 at −30 mV [77]*	*Open probability:* *0.21 at 50 mV to 0.57 at −50 mV [32]*
***TASK-3***	18 pS in 140 mM KCl (pipette) [78] 17–27 pS in 140 mM KCl [79]	12–83 pS in 150 mM KCl [33]
***HCN***	~1 pS for I_f_ in 5.4 (bath)/70 (pipette) mM KCl [80] 0.46 and 1.71 pS for HCN1 and HCN2, respectively (in 110 mM KCl) [81]	Not determined at single channel level (for whole-mitoplast recording, see [34]

As mentioned above, all these channel activities mediate influx of K^+^ into the matrix. This K^+^ flux in the presence of permeable anions (e.g., inorganic phosphate) takes place along with osparison of single-channel conductance values ofmotically obligated water and therefore results in mitochondrial swelling. Therefore, to control K^+^ concentration, a K^+^/H^+^ exchanger ensures exit of K^+^ in the so-called K^+^ cycle [82]. The molecular identity of this exchanger remains debated, even though convincing evidence has been accumulated in favor of the hypothesis that envisions LETM1 as the K^+^/H^+^ exchanger (for a recent review see [83]). LETM1 has originally been identified as a protein linked to mitochondrial K^+^ homeostasis [84], but was later proposed to be the long-sought electroneutral calcium/proton antiporter (2H^+^/Ca^2+^) of the IMM [85,86]. However, a more recent work provided compelling evidence, using a novel potassium probe mitoPOP able to monitor the mitochondrial K^+^ concentration, that (i) LETM1 deletion caused K^+^ accumulation in the mitochondrial matrix; (ii) LETM1, able to transport both K^+^ and Na^+^, regulated mitochondrial calcium fluxes in a sodium-dependent manner [87]. Thus, in the so-called calcium cycle, LETM1 would exert a regulatory effect on calcium exit through the Na^+^ /Ca^2+^ antiporter, whose function would be linked to the extrusion of matrix Na+ via LETM1. Unfortunately, more recent works [88,89] did not consider this possibility when interpreting experimental results. Independently of the nature of the transported ions, LETM1 remains an important player in mitochondrial biology, as it has recently been shown to cause cristae-like invaginations even in artificial liposomes, raising the possibility that this transporter contributes to cristae shaping [90]. A previous work linked LETM1 function to mitochondrial fragmentation, but observed no changes in mitochondrial morphology in fibroblasts from Wolf–Hirschhorn syndrome patients, in which monoallelic LETM1 deletion occurs [91].

## 3. The Mitochondrial ATP-Dependent Potassium Channel(s)

In addition to uncertainties regarding LETM1, the field of mitochondrial potassium-transporting proteins has to deal with the molecular identification of one of the most important and most well-known activities, the elusive mitochondrial ATP-dependent potassium channel (KATP). KATP exerts crucial function in the PM, for example by regulating insulin secretion [92]. The PM channel comprises four channels forming subunits Kir6.1 or Kir6.2 (encoded by *KCNJ8* and *KCNJ11*, respectively) and of four regulatory SUR subunits (SUR1, SUR2A/SUR2B, encoded by *ABCC8* and *ABCC9*, respectively), which act as sulphonylurea receptors. The association of a particular SUR with a specific Kir6.x subunit constitutes the ATP-dependent K^+^ current (KATP) in each tissue (for reviews, see e.g., [92,93]. Soon after the first report that applied the patch clamp technique to mitoplasts obtained by osmotic swelling and rupture of the OMM [94], Inoue and colleagues identified, using the same technique, a channel in the IMM that was inhibited by ATP, and was therefore named as mitoKATP [25].

The conductance of KATP of the PM ranges from 33–35 pS for the channels composed of Kir6.1 to 67–80 pS for those constituted by Kir6.2, in symmetrical 140 mM KCl (for review see e.g., [18]). However, conductance as low as 13 pS (in 140 mM symmetrical KCl solution) was recorded in rat mesenteric artery vascular smooth muscle cells (VSMC) [51]. In this latter cell type, the diversity of molecular entities of KATP channels is illustrated by their single-channel conductance ranging from 13 to 135 pS, with distinct conductance values of 13, 20, 50, 111 and 135 pS, recorded under similar ionic conditions in various studies [51,95].

Attribution of these channel activities with different conductance values to KATP in most experiments is based on the pharmacological profiling of channel activities. The hallmarks of PM KATP comprise inhibition by ATP/Mg^2+^ or glibenclamide and activation by P-1075, BMS 191095, cromakalim, pinacidil and nicorandil [96,97,98,99,100,101,102]. Therefore, these drugs have been tested on mitochondrial K^+^ channel activities by different groups. In addition, diazoxide was identified as an agent that activates mitoKATP more efficiently than PM KATP, and HMR 1098 was proposed as a specific inhibitor of PM KATP but not of mitoKATP [103,104].

The above pharmacological drugs were then exploited in different cell types to measure mitoKATP activity in mitoplasts directly by patch clamp. As mentioned above, in the first study, a ~10 pS channel was identified as mitoKATP [25], but in subsequent studies, different conductance values were observed. Table 2 summarizes all data published to date, in which the main biophysical and pharmacological properties of mitoKATP activity in the native IMM were determined using the patch clamp technique. Although in two studies (in Jurkat [56] and in rat liver mitochondria [25]) an ATP-sensitive channel with low conductance was observed (15 and 9.7 pS in 100/33 mM KCl or symmetrical 150 mM KCl, respectively), in other works, the conductance reached ~100 pS (in 150 mM KCl) [32,57,105]. Thus, the conductance values around 100 pS recorded in native mitochondrial membranes are compatible with the ones observed for PM KATP formed by Kir6.2-SUR2A complexes [53,54]. This finding, along with the pharmacological profile mentioned above, prompted the researchers to propose the Kir6.2 inward rectifying channel as the main pore-forming constituent of mitoKATP.

**Table 2:** Biophysical characteristics of mitoKATP channel activities recorded by patch clamping of the inner mitochondrial membrane (excised inside-out configuration). “+” and “−” denote activation or inhibition of mitoKATP, respectively.

Tissue/Cell Origin	of	Method of Mitoplast	Recording Medium		Single-Channel	Modulation	Reference
		Preparation			Conductance		
Rat liver		Giant mitoplasts obtained by digitonin–swelling fusion	Pipette: 100 mM KCI, 7.5 mM sodium-MOPS, pH 7.2, 1 mM EGTA, 0.55 mM CaCl_2_ Bath: 33.3 mM KCI. 66.7 mM NaCI, 7.7 mM Na-MOPS, pH 7.2, 2 mM EGTA		9.7 pS at negative membrane potentials	ATP 2 mM (−) 4-aminopyridine −*5* mM (−) glybenclamide *5* μM (−)	[25]
Jurkat lymphocyte	T	Swelling	Pipette/bath: 150 mM KCl, 10 mM HEPES and 100 (or 200) μM CaCl_2_ (pH = 7.2)		15 and 82 pS at negative and positive potentials, respectively	5-hydroxydecanoic acid 1 mM (−) nitric oxide 2 μM (−) ATP 0.5–25 mM (−)	[56]
Human dermal fibroblast		Swelling	Pipette/bath: 150 KCl, 10mM HEPES, and 200 CaCl_2_ at pH 7.2.	mM μM	100 pS	1 mM Mg^2+^ plus 500 μM ATP (−) diazoxide 30 μM (+) BMS 191,095 10 μM (+) glibenclamide 30 μM (−) 5-hydroxydecanoic acid 150 Μm (−)	[105]
Heart-derived H9c2 cells and H9c2 ROMK2		Swelling	Pipette/bath: 150 mM of KCl, 10 mM of HEPES, and 200 μM of CaCl_2_ at pH = 7.2		94–97 pS	5-hydroxydecanoic acid 100 μM (−) tertiapin Q 100 nM (−)	[32]
Overexpressing cells						1 mM Mg^2+^ plus 500 μM ATP (−) diazoxide (+) glibenclamide 50 μM (−, partial inhibition)	
Primary Human dermal fibroblasts		Swelling	Pipette/bath: 150 mM of KCl, 10 mM of HEPES, and 200 μM of CaCl_2_ at pH = 7.2		100 pS	naringenin (Nar) 10 μM (+) diazoxide 30 μM (+) 5-hydroxydecanoic acid 500 μM (plus Nar) (−) glibenclamide 10 μM (plus Nar) (−)	[57]

Despite the fact that the molecular composition of mitoKATP was not elucidated, the field of mitoKATP channel underwent a rapid evolution when pharmacological studies highlighted an important role of this channel in cardioprotection, in particular in attenuating the damage caused by ischemia- reperfusion (for recent reviews see, e.g., [6,7,13]. Moreover, mitoKATP activation has been proposed to exert neuroprotective effects [106] and to modulate mitochondrial dynamics, biogenesis and neurodegenerative disorders such as Parkinson [107]. Interestingly, diazoxide, the activator of mitoKATP, was shown to improve memory in a mouse model of Alzheimer’s disease and ameliorate amyloid-β and tau pathologies [108]. Neuronal injury was also attenuated in models of the metabolic disease methyl-malonic acidemia by mitoKATP openers [109].

Most of the above studies underlining the patho-physiological role of mitoKATP were carried out with diazoxide as a channel activator. However, this drug also exerts K^+^ channel-independent effects, such as inhibition of complex II of the respiratory chain (succinate dehydrogenase) and uncoupling action, leading researchers to question the role of mitoKATP in ischemic preconditioning [110]. Moreover, diazoxide seems to also have an impact on the expression of some proteins, as it upregulates the two components of the calcium-release-activated calcium channel (I_CRAC_, also called the SOCE channel), STIM1 and Orai1 in cardiomyocytes [111]. The antioxidant *N*-acetyl cysteine (NAC), 5-hydroxydecanoate (5-HD), and the MAPK pathway inhibitor UO126 were able to attenuate diazoxide-induced upregulation of STIM1 and Orai1 expression, suggesting that an ROS- and MAPK-dependent pathway is activated by this mitoKATP opener. The authors hypothesized that alteration of the distribution pattern of STIM1, causing decreased Ca^2+^ influx into the cells, may contribute to cardioprotection against ischemic insults. However, direct electrophysiological evidence that I_CRAC_ activity is decreased in cells incubated with diazoxide has not been reported.

**Figure 1:**
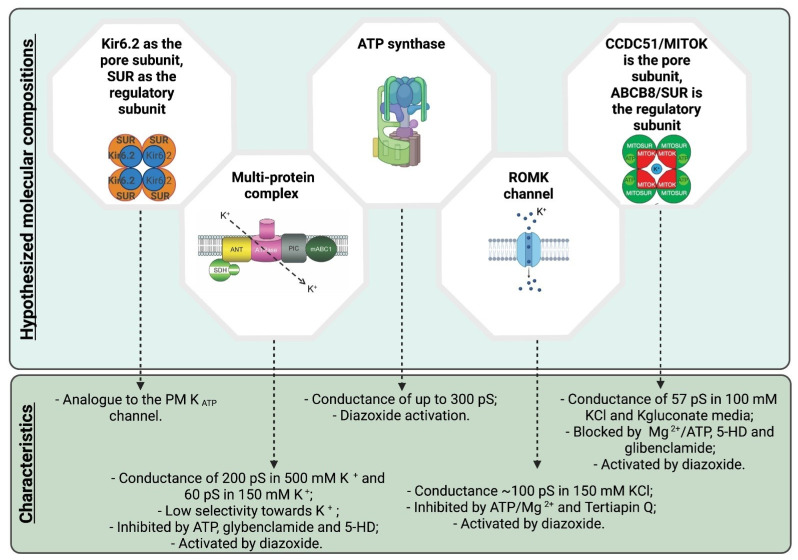
Schematic summary of the various hypotheses for the molecular composition of mitoKATP (see text for details). Created with BioRender.com (accessed on 22 June 2021).

Thus, while the protective effect of diazoxide against ischemic damage has been confirmed in several studies, the specificity of action via mitoKATP channels could not be proven in the absence of the molecular identity of this channel. As mentioned above, based on the conductance values observed in native IMM and by analogy with the PM KATP channel, Kir6.2 was first proposed as the pore-forming component, while SUR subunits were proposed as regulatory components of this complex (Figure 1). However, mito KATP channel activity, determined using the thallium (Tl^+^) flux assay in mitochondria isolated from WT or Kir6.2^−/−^ littermate hearts was identical, even though the channel was required for the action of diazoxide to promote ischemic preconditioning. The authors therefore concluded that Kir6.2 is not a component of mitoKATP [112]. Direct electrophysiological recordings on mitoplasts from Kir6.2^−/−^ animals would be useful to further strengthen such a conclusion.

As an alternative to the Kir6.2 hypothesis, a multi-protein complex comprising complex II (succinate dehydrogenase), complex V (ATP synthase), an ATP-binding cassette protein 1 (mABC1), the phosphate carrier and the adenine nucleotide translocator (ANT) [46] was put forward. This multi-protein complex was incorporated into proteoliposomes and was characterized using the planar lipid bilayer electrophysiological technique, revealing a passage of K^+^, giving rise to a conductance of 200 pS in 500 mM K^+^ (Hepes was used as a counterion). Interestingly, the observed activity showed low selectivity towards potassium but was inhibited by ATP, glybenclamide, or 5-HD, even in the presence of the activator diazoxide, as one would expect for mitoKATP. Unfortunately, mass spectrometry analysis was not provided on the complex isolated from the IMM, thus it cannot be excluded that other protein(s) present in the preparation are responsible for the observed channel activity. It is of note that ANT and the phosphate carrier can form ion channels on their own (for detailed review see e.g., [18]) and ANT was shown to also mediate proton leak [113]. In addition, highly purified complex V also forms channels under certain conditions (in a Ca^2+^-dependent way) with characteristics resembling the permeability transition pore (PTP) [114]. However, the described conductance values of ANT, complex V and the phosphate carrier measured in K^+^-based medium are different from those observed in the study of Ardehali and colleagues.

Interestingly, a recent study re-proposed the participation of complex V in mitoKATP formation [115]. In particular, isolated ATP synthase (purity was, however, not assessed using mass spectrometry) was studied under ionic conditions that are physiologically relevant (with a ratio of 10^6^:1 between K^+^ and H^+^), and so in the presence of both protons and potassium ions. Based on electrophysiological experiments, the authors concluded that the ATP synthase allows the passage of K^+^ in addition to protons (a unitary conductance of up to ~300 pS was observed) and underlies the so-called K^+^ uniporter (mitoKATP). Interestingly, diazoxide in this context would act as an activator of mitoKATP by directly binding to the inhibitory protein of complex V, IF1 [116]. The proposal that complex V is responsible also for the formation of the PTP makes it all quite intriguing. Altogether, the authors proposed that the increase in ATP synthesis guided by K^+^ and H^+^ would allow complex V to operate as a primary mitochondrial uniporter of K^+^ that regulates the match between energy supply and demand, and as the recruitable mitochondrial KATP channel that can limit ischemia-reperfusion injury. While genetic deletion or point mutants of certain subunits of the ATP synthase were shown to alter PTP properties in classical biochemical and electrophysiological assays [117,118,119,120], efforts to specifically study (diazoxide-dependent) K^+^ transport across genetically modified ATP synthase complexes have not been taken into consideration up to date.

In parallel with the above studies, research also focused on the renal outer medullary kidney channel (ROMK2) as a possible mitoKATP channel forming protein. ROMK2 is a short, mitochondria-targeted isoform of ROMK (also called Kir1.1, encoded by *KCNJ1*) which was shown to co-localize with mitochondrial ATP synthase in cardiomyocytes [48]. ROMK itself is ATP-sensitive and can associate with SUR2B. The single-channel conductance of PM ROMK at negative voltages is 32–43 pS (with 110 mM KCl in the pipette). In the work of Foster and colleagues [48], short hairpin RNA-mediated knockdown of ROMK inhibited the ATP-sensitive, diazoxide-activated component of mitochondrial thallium uptake, used as a proxy for potassium uptake into the matrix. Importantly, tertiapin Q, a venom toxin-derived inhibitor of ROMK channels, almost completely abolished thallium uptake. Moreover, the expression level of ROMK2 in the heart-derived cell line H9C2 correlated well with sensitivity to cell death triggered by oxidative stress (via application of tertbutyl hydroperoxide (tBHP)). Thus, these results strongly suggest that ROMK2 might correspond, at least in some tissues, to mitoKATP. Electrophysiological evidence in favor of this hypothesis was provided more recently by the group of Adam Szewczyk, where patch clamp on dermal fibroblast mitochondria revealed a 100 pS (in symmetric 150 mM KCl) channel activity that was ascribed to ROMK and was activated by diazoxide while suppressed by ATP/Mg^2+^, glibenclamide and 5-hydroxydecanoic acid [105]. Moreover, genetic overexpression of ROMK2 in heart-derived H9c2 cells enhanced mitoKATP activity directly recorded in the IMM by patch clamp. This channel displayed the typical biophysical and pharmacological characteristics of mitoKATP (~100 pS in 150 mM KCl, inhibition by ATP/Mg^2+^, activation by diazoxide) and was inhibited by Tertiapin Q [32]. On the other hand, the observation that recombinant ROMK2 incorporated into nanodiscs shows a chord conductance of only 10 pS in 50/150 mM KCl, is intriguing [121]. Further work is required to find the reason for this difference in the biophysical properties of the recombinant versus native ROMK2 channels. While the electrophysiological and pharmacological properties of ROMK2 altogether are compatible with this protein being mitoKATP, a recent study using genetic, global or cardiomyocyte-specific knockout mice for ROMK2 discovered that isolated mitochondria from the latter mice still showed swelling upon addition of the mitoKATP opener BMS-191095 and were characterized by unchanged matrix volume responses during oxidative phosphorylation [122].

ROMK-less heart mitochondria exhibited a decreased threshold for calcium-triggered PTP opening but molecular details of how the presence of ROMK can de-sensitize PTP towards a calcium increase are missing: the association of ROMK2 with the ATP synthase (see above) might contribute to this effect. Alternatively, mitoKATP opening may slightly depolarize the IMM and reduce the driving force for Ca^2+^ entry, thereby counteracting mitochondrial Ca^2+^ overload and subsequent mPTP opening [123]. Altogether, the authors of this seminal work concluded that cardiomyocyte ROMK is not a major component of the cardioprotective mitoKATP channel, although to our knowledge the possibility that other Kir channel-forming subunits undertook the function of ROMK in the KO heart has not been ruled out.

As a last chapter in the “saga” of mitoKATP identification, a mitochondria- specific, ubiquitously expressed coiled-coil domain containing protein, CCDC51, was proposed to form a K^+^-permeable monovalent cationic pore [124]. The channel formed by recombinant CCDC51 was inhibited by ATP, glibenclamide and 5-hydroxydecanoate and was activated by diazoxide, only when co-assembled with a mitochondrial ABC protein (ABCB8). The conductance of the CCDC51–ABCB8 complex, reconstituted in proteoliposomes and studied by electrophysiology (planar lipid bilayer), was 57 pS in 100 mM K-gluconate medium. Importantly, using cells where CCDC51 was genetically deleted, it has been demonstrated that this protein controls mitochondrial volume and efficiency of oxidative phosphorylation, as expected for mitoKATP. Moreover, diazoxide-triggered 86Rb^+^ (used as surrogate of K^+^) influx into isolated energized liver mitochondria was completely abolished in CCDC51-less organelles, suggesting that at least in the liver, diazoxide does not trigger K^+^ uptake by acting on ATP synthase or on the complex comprising complexes II and V, ANT, mABC1 and the phosphate carrier or on Kir6.2/Kir6.1 channels. Most importantly, the whole-body deletion of CCDC51 almost completely suppressed the cardioprotection that was elicited by the pharmacological preconditioning induced by diazoxide, suggesting again a crucial role for CCDC51 (in complex with ABCB8) in the protective action of diazoxide. In order to correlate these results with mitoKATP channel activities described so far in the IMM from different tissues, it will be very important to characterize by patch clamp the ATP- and diazoxide-dependent activities in the IMM of mitochondria from different CCDC51-KO mouse tissues (a work that is under way in our laboratory).

As a future perspective, one may envision a collaborative effort among chemists, cell biologists, electrophysiologists and cardiologists, to understand if mitoKATP composition can vary depending on the tissue type. A recent study described the synthesis of a mitochondriotropic, triphenylphosphonium-linked [125] variant of the mitoKATP activator spirocyclic benzopyrane F81 and showed that mito-F81 exerted cardioprotective effects at a 10-fold lower dose with respect to the parent compound [126]. Unfortunately, this study did not directly test the inhibitory effect of the modified compound on mitoKATP in electrophysiological experiments nor compared EC_50_ values of the two compounds for inhibition of Tl+ uptake. Nonetheless, this compound has the advantage of most probably acting prevalently on mitoKATP (and not on PM KATP) and could be used in various cell lines knocked-out for Kir6.2, ROMK, CCDC51, and ANT, as well as in various ATP synthase subunit-knocked down cells. In addition, this compound could also be used for affinity chromatography followed by mass spectrometry to identify the proteins interacting with high affinity. On the other hand, caution should be taken, as TPP^+^-targeting may modify the original properties of chemicals, decrease their affinity for the target (e.g., [127]) and trigger additional and/or off-target effects as well (e.g., [43,128]).

## 4. Future Outlook

In summary, while huge progress has been made in mitochondrial potassium channels in the last few decades, many questions remain open. A cross-disciplinary effort will likely resolve the mysteries regarding possible plasticity in channel formation, dual (or even multiple) targeting to intracellular membranes, and pharmacological regulation. In addition, recent studies highlighted a differential role for some PM and mitochondrial channels, exploiting mitochondria-targeted drugs. While a thorough characterization of these drugs is mandatory, they may represent a handy pharmacological tool against various diseases (see, e.g., [126,127,129]).

Administration of organelle-specific drugs is the modern trend to achieve significant therapeutic effects (e.g., [130]). However, the problem of possible off-target effects is certainly the barrier that must be overcome in molecular pharmacology to achieve selective targeting and accumulation in mitochondria [5,131]. In the past few decades, various strategies have been developed to target drugs into the mitochondria. Such strategies involve direct conjugation of a targeting ligand to drugs and/or attachment of the targeting ligand to a nanocarrier [132]. The direct conjugation of the ligand is certainly a valid approach through which drugs can easily reach the mitochondria—it is the simplest and easiest method; however, the conjugation procedure can decrease the biochemical effects within the mitochondria. In the nanocarrier system, on the other hand, the therapeutic effect is not lost because the problem of physical interaction and solubility is solved, but optimization is still a challenge due to the use of many different possible compositions to identify the best nanocarrier. Finally, the administration of thermoresponsive drugs to the mitochondria for cancer therapy has recently been described [133].

Mitochondria-targeted drugs have been extensively studied in clinical applications, and several formulations have been approved by the US Food and Drug Administration and European Medicines Agency for clinical applications in patients with cancer. A brief overview of some major approaches aiming to modulate the Krebs cycle, electron transport chain, anaplerosis, mitoROS release and mitochondria-driven apoptosis in cancer is given in e.g., [134].

In terms of future perspectives, hopefully mitochondrial ion channels will become preferred targets not only in the context of cancer but also of other human diseases. To our knowledge, to date, no drugs specifically targeting these channels are in clinical use.
